# Supplementary material for: Investigating Markers of the NLRP3 Inflammasome Pathway in Alzheimer’s Disease: A Human Post-Mortem Study
Source: Genes (Basel). 2021 Oct 30;12(11):1753. doi: 10.3390/genes12111753 (PMC8622528; doi:10.3390/genes12111753)
Supplement: Supplementary file 1 [file genes-12-01753-s001.zip › Supplementary figure S3.pdf]

Supplementary Figure S3

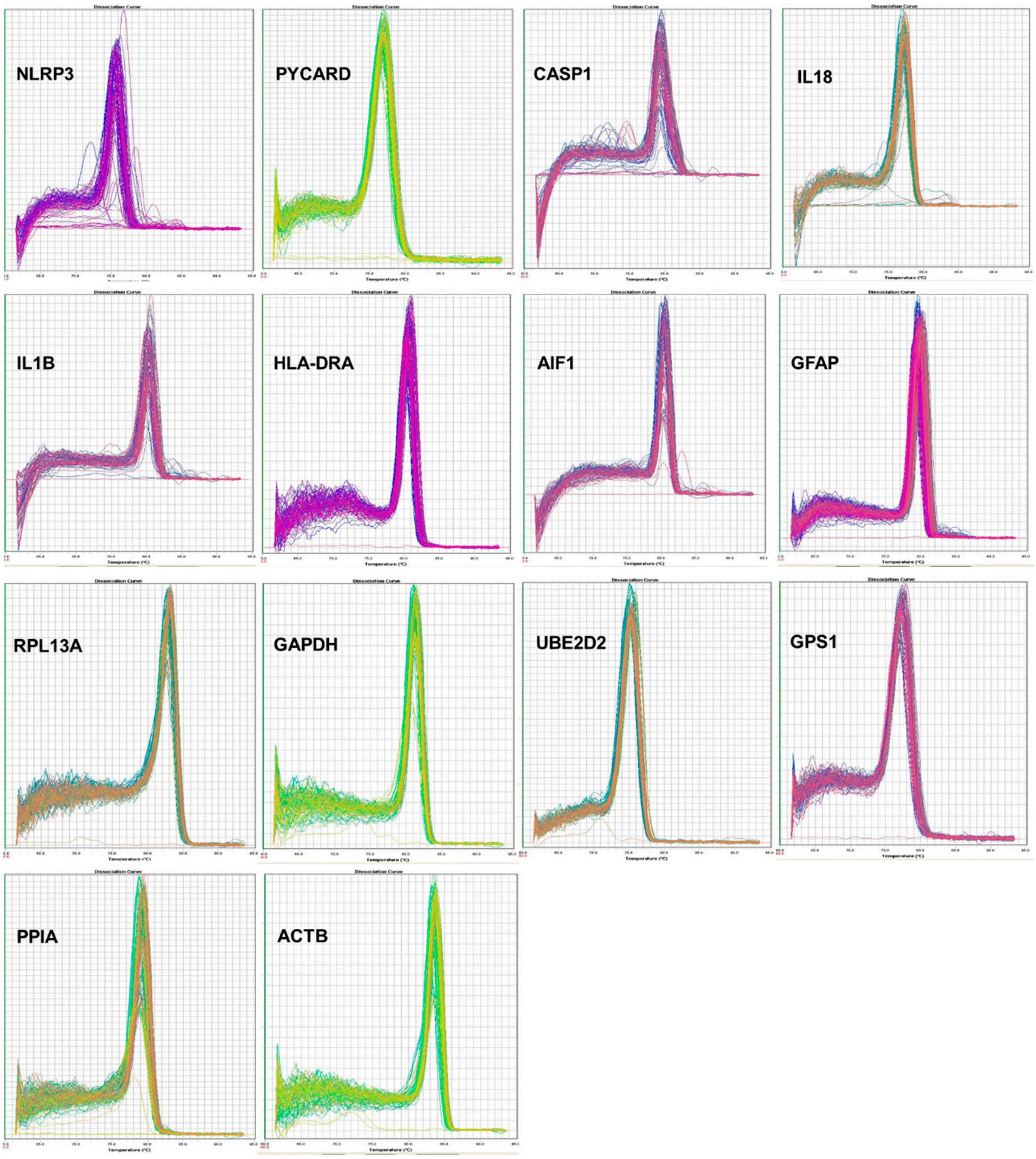

Figure S3: Melting curves for all analyzed candidate reference genes and the genes of interest.
