# Supplementary material for: Investigating Markers of the NLRP3 Inflammasome Pathway in Alzheimer’s Disease: A Human Post-Mortem Study
Source: Genes (Basel). 2021 Oct 30;12(11):1753. doi: 10.3390/genes12111753 (PMC8622528; doi:10.3390/genes12111753)
Supplement: Supplementary file 1 [file genes-12-01753-s001.zip › Supplementary table S1.pdf]

Supplementary Table S1

Table S1: MIQE checklist for RT-qPCR experiment.

ITEM TO CHECK	IMPORTANCE	COMMENTS
**EXPERIMENTAL DESIGN**		
Definition of experimental and control groups	E	Materials & Methods; Table 1
Number within each group	E	40 AD & 20 controls
Assay carried out by core lab or investigator's lab?	D	Investigator’s lab
Acknowledgement of authors' contributions	D	Yes
**SAMPLE**		
Description	E	Frozen postmortem brain tissue
Volume/mass of sample processed	D	40-60 mg
Microdissection or macrodissection	E	Macro
Processing procedure	E	Materials & Methods
If frozen - how and how quickly?	E	Frozen at -80°C after post-mortem delay; Table 1
If fixed - with what, how quickly?	E	Unfixed
Sample storage conditions and duration	E	Stored at -80°C in brain bank of Salford Royal Foundation Trust hospital
**NUCLEIC ACID EXTRACTION**		
Procedure and/or instrumentation	E	Materials & Methods
Name of kit and details of any modifications	E	TRIzol™ Reagent (Thermo Fisher) & Monarch® RNA Cleanup Kit (New England Biolabs) ; Materials & Methods
Source of additional reagents used	D	Materials & Methods
Details of DNase or RNAse treatment	E	ND; DNA contamination is tested & primers are exon junction span

Contamination assessment (DNA or RNA)	E	DNA contamination assessment by qPCR without RT step
Nucleic acid quantification	E	RNA quantification performed
Instrument and method	E	NanoDrop™ (Thermo Fisher Scientific Inc)
Purity (A260/A280)	D	NanoDrop™ (Thermo Fisher Scientific Inc)
Yield	D	NanoDrop™ (Thermo Fisher Scientific Inc)
RNA integrity method/instrument	E	RNA integrity number (RIN) was assessed by Agilent 2200 TapeStation System
RIN/RQI or Cq of 3' and 5' transcripts	E	RIN was assessed
Electrophoresis traces	D	Native agarose gel electrophoresis traces
Inhibition testing (Cq dilutions, spike or other)	E	Serial dilution was performed
**REVERSE TRANSCRIPTION**		
Complete reaction conditions	E	Materials & Methods
Amount of RNA and reaction volume	E	500 ng of RNA in a 20 µl reaction
Priming oligonucleotide (if using GSP) and concentration	E	2X RT Reaction Mix includes oligo(dT)20 (2.5 µM) & random hexamers (2.5 ng/µL)
Reverse transcriptase and concentration	E	Invitrogen SuperScript III First-Strand Synthesis Supermix for qRT-PCR
Temperature and time	E	According to manufacturer’s instructions
Manufacturer of reagents and catalogue numbers	D	SuperScript III First-Strand Synthesis Supermix for qRT-PCR / Thermo Fisher Cat No.11752-050
Cqs with and without RT	D*	See Results for details
Storage conditions of cDNA	D	cDNA stored at -20°C
**qPCR TARGET INFORMATION**		
If multiplex, efficiency and LOD of each assay	E	Not multiplex. PCR efficiencies were given in Results
Sequence accession number	E	Yes
Location of amplicon	D	Table 2

Amplicon length	E	Confirmed by electrophoresis
In silico specificity screen (BLAST, etc)	E	Yes
Pseudogenes, retropseudogenes or other homologs?	D	NA
Sequence alignment	D	NA
Secondary structure analysis of amplicon	D	NA
Location of each primer by exon or intron (if applicable)	E	Table 2
What splice variants are targeted?	E	Table 2
**qPCR OLIGONUCLEOTIDES**		
Primer sequences	E	Table 2
RTPrimerDB Identification Number	D	NA
Probe sequences	D**	NA
Location and identity of any modifications	E	None
Manufacturer of oligonucleotides	D	Thermo Fisher
Purification method	D	Desalted
**qPCR PROTOCOL**		
Complete reaction conditions	E	Materials and Methods
Reaction volume and amount of cDNA/DNA	E	10 µl reaction with 1 µL cDNA
Primer, (probe), Mg++ and dNTP concentrations	E	0.25 µM each primer
Polymerase identity and concentration	E	Power SYBR™ Green PCR Master Mix (Thermo Fisher)
Buffer/kit identity and manufacturer	E	Power SYBR™ Green PCR Master Mix (Thermo Fisher)
Exact chemical constitution of the buffer	D	Power SYBR™ Green PCR Master Mix (Thermo Fisher)
Additives (SYBR Green I, DMSO, etc.)	E	Power SYBR™ Green PCR Master Mix (Thermo Fisher)

Manufacturer of plates/tubes and catalog number	D	Applied Biosystems MicroAmp Optical 384-well reaction plate. Cat No. 4309849
Complete thermocycling parameters	E	Materials and Methods
Reaction setup (manual/robotic)	D	Manual
Manufacturer of qPCR instrument	E	7900HT Real-Time PCR System (Thermo Fisher)
**qPCR VALIDATION**		
Evidence of optimisation (from gradients)	D	Optimisation was performed using pool cDNA
Specificity (gel, sequence, melt, or digest)	E	Confirmed by gel electrophoresis and melting curve
For SYBR Green I, Cq of the NTC	E	NTC Cq >40
Standard curves with slope and y-intercept	E	Table 2
PCR efficiency calculated from slope	E	Yes, see Table 2
Confidence interval for PCR efficiency or standard error	D	NA
r2 of standard curve	E	approximately 0.99
Linear dynamic range	E	NA
Cq variation at lower limit	E	ND
Confidence intervals throughout range	D	ND
Evidence for limit of detection	E	NA
If multiplex, efficiency and LOD of each assay	E	Not multiplex
**DATA ANALYSIS**		
qPCR analysis program (source, version)	E	7900HT Real-Time PCR System Software
Cq method determination	E	Automatic baseline setting and manual threshold setting
Outlier identification and disposition	E	Yes
Results of NTCs	E	>40 cycles

Justification of number and choice of reference genes	E	Two reference gene transcripts used. Validation by RefFinder ranking of the six candidates
Description of normalisation method	E	2-ΔΔCt method by normalisation against RPL13A and GAPDH
Number and concordance of biological replicates	D	Materials and Methods
Number and stage (RT or qPCR) of technical replicates	E	Materials and Methods
Repeatability (intra-assay variation)	E	Standard deviation of triplicates <0.2
Reproducibility (inter-assay variation, %CV)	D	Analysed with raw Cq value of RGs.
Power analysis D	D	NA
Statistical methods for result significance	E	Materials and Methods

* Assessing the absence of DNA using a no RT assay is essential when first extracting RNA. Once the sample has been validated as DNA-free, inclusion of a no-RT control is desirable, but no longer essential. ** Disclosure of the probe sequence is highly desirable and strongly encouraged. E = essential information; D = desirable information; NA = not applicable; ND = not done.
