# Supplementary material for: Investigating Markers of the NLRP3 Inflammasome Pathway in Alzheimer’s Disease: A Human Post-Mortem Study
Source: Genes (Basel). 2021 Oct 30;12(11):1753. doi: 10.3390/genes12111753 (PMC8622528; doi:10.3390/genes12111753)
Supplement: Supplementary file 1 [file genes-12-01753-s001.zip › Supplementary table S2.pdf]

Supplementary Table S2

Table S2. Overview of genes and corresponding primer pairs for reverse transcriptase quantitative real-time PCR.

Gene symbol	Accession no.	Primer sequence (5′-3′)		Location of amplicon	Exon junction span	Bp	E (%)	R^2	Slope	Ta
**Reference Genes**										
ACTB	NM_001101	Sense:<br>Anti-sense:	AGAGCTACGAGCTGCCTGAC<br>AGCACTGTGTTGGCGTACAG	797……816<br>980……961	Yes	184	97.7	0.990	-3.377	65.8
RPL13	NM_012423	Sense:<br>Anti-sense:	AGCCTACAAGAAAGTTTGCCTAT<br>TCTTCTTCCGGTAGTGGATCTTGGC	411…….433<br>536……512	Yes	126	98.7	0.996	-3.354	63.3
PPIA	NM_021130	Sense:<br>Anti-sense:	TCCTGGCATCTTGTCCATG<br>CCATCCAACCACTCAGTCTTG	326……344<br>415……395	Yes	90	96.6	0.963	-3.406	62.7
UBE2D2	NM_003339	Sense:<br>Anti-sense:	TGCCTGAGATTGCTCGGATCT<br>TCGCATACTTCTGAGTCCATTCC	812……832<br>892……870	Yes	81	105.5	0.993	-3.197	65.0
GADPH	NM_002046	Sense:<br>Anti-sense:	CCTGCACCACCAACTGCTTA<br>GTCTTCTGGGTGGCAGTGATG	528……547<br>636……616	Yes	109	105.9	0.994	-3.189	65.7
GPS1	NM_212492	Sense:<br>Anti-sense:	CGTGCCCTCATCCAGTATTTC<br>TCCTATGCATGTCGGCTGAC	1664……1684<br>1715……1696	Yes	52	86.4	0.994	-3.697	63.8
**Targeted genes in the NLRP3 system**										
NLRP3	NM_004895	Sense:	GATCTTCGCTGCGATCAACAG	965……985	Yes	81	105.2	0.999	-3.203	64.2

		Anti-sense:	CGTGCATTATCTGAACCCCAC	1045……1025						
PYCARD	NM_145182	Sense:<br>Anti-sense:	ATCCAGGCCCCTCCTCAGTC<br>TTGTGACCCTCGCGATAAGC	368……387<br>452……433	Yes	85	93.6	0.999	-3.485	65.4
CASP1	NM_001257118	Sense:<br>Anti-sense:	GAAGGCATTTGTGGGAAGAA<br>CATCTGGCTGCTCAAATGAA	752……771<br>1175……1156	Yes	424	92.2	0.998	-3.524	61.6
IL18	NM_001562	Sense:<br>Anti-sense:	CGCTTCCTCTCGCAACAAAC<br>ATTCCAGGTTTTCATCATCTTCAGC	158……177<br>300……276	Yes	143	105.0	0.986	-3.208	64.7
IL 1B	NM_000576	Sense:<br>Anti-sense:	GGCTGCTCTGGGATTCTCTTC<br>ATAAGCCATCATTTCACTGGCG	32……52<br>129……108	Yes	98	91.3	0.999	-3.549	64.3
**Glial activation markers**										
HLA-DRA	NM_019111	Sense:<br>Anti-sense:	GACAAGTTCACCCCACCAGT<br>ACTTGCGGAAAAGGTGGTCT	467……486<br>581……562	No	115	106.9	0.999	-3.165	65.2
AIF1	NM_001623	Sense:<br>Anti-sense:	TGTCTCCCCACCTCTACCAG<br>CATCCAGCCTCTCTTCCTGC	64......83<br>172......153	Yes	109	99.66	0.977	-3.330	65.3
GFAP	NM_002055	Sense:<br>Anti-sense:	CCCTCACCAAATTCCACCCG<br>TTGAGTTGCAGGTAGCAGGG	3138……3157<br>3228……3209	No	91	109.4	0.995	-3.116	65.2

Bp: length of the amplicon in base pair; E: amplification efficiency; R^2: coefficient of correlation; Ta: annealing temperature.
