# Supplementary material for: Investigating Markers of the NLRP3 Inflammasome Pathway in Alzheimer’s Disease: A Human Post-Mortem Study
Source: Genes (Basel). 2021 Oct 30;12(11):1753. doi: 10.3390/genes12111753 (PMC8622528; doi:10.3390/genes12111753)
Supplement: Supplementary file 1 [file genes-12-01753-s001.zip › Supplementary table S3.pdf]

## Supplementary Table S3

**Table S3.** Summarize of reference gene stability in studies that assessed reference gene stability in post-mortem brain of neurodegenerative diseases.

| Most stable RGs | Less stable RGs | Sample size | Brain region | Additional information of RNA | Method | Reference |
|---|---|---|---|---|---|---|
| GAPDH | ACTB, 18SrRNA | AD = 13<br>NC = 6 | Frontal cortices | Brain weight, PMI, Age at death<br>PMI and age at death do not influence mRNA expression | Delta CT | [1] |
| ACTB<br>GUS | TBP, B2M, PGK, huPO, CYC, GAPDH, HPRT, TfR | AD = 16<br>PD = 3<br>DLB = 8<br>NC = 6 | Frontal cortex | Agonal state, PMI, Brain pH | Delta CT | [2] |
| Medial Temporal Gyrus<br>AD:UBC, HMBS, SDHA,YWHAZ<br>PD: GAPDH, SDHA, HMBS<br>DLB:B2M, SDHA,GAPDH<br>Cerebellum<br>AD:YWHAZ, GAPDH, B2M<br>PD:HMBS, GAPDH, SDHA | TBP, UBC, YWHAZ,RP II, HMBS, SDHA | AD = 6<br>PD = 6<br>DLB = 6<br>NC = 5 | Medial temporal gyrus<br>cerebellum | No correlation between RIN and disease progression nor gene expression | geNorm | [3] |

| | | | | | | |
|---|---|---|---|---|---|---|
| DLB:UBC, GAPDH, B2M | | | | | | |
| PPIA<br>UBE2D2<br>GPS1 | PGK1, GAPDH, GUSB,<br>M-RIP, POL2RF,<br>YES1, ACTB | AD = 9<br>NC= 11 | Frontal cortex | PMD<br>RIN | geNorm<br>NormFinder<br>BestKeeper | [4] |
| XPNPEP1 | ATP5E, AARS,<br>GAPVD1, CSNK2B,<br>OSBP, NAT5, DCTN2,<br>BECN1, GAPDH,<br>QARS, TUBB | AD =11 NC =7<br>ALS = 10 NC = 10<br>HTD = 9 NC = 10<br>MS = 10 NC = 10<br>PD = 12 NC = 8<br>SCZ = 10 NC = 10 | Entorhinal cortex<br>Cervical spinal cord<br>Ventral head of caudate nucleus<br>Superior frontal gyrus<br>Substantia nigra<br>Temporal cortex left BA22 | RIN | Ranking by<br>coefficient of<br>variance (CV) data<br>based on microarray<br>and RT-PCR | [5] |
| Prefrontal cortex<br>AD: UBE2D2, RPL13, PPIA<br>PD:UBE2D2, RPL13,PUM1<br>Cerebellum<br>AD: UBE2D2, RPL13, TBP<br>PD: UBE2D2, PPIA, ATP5B | B2M, UBC, ACTB,<br>EIF4A2, GADPH,TOP1 | AD = 10<br>PD = 10<br>MSA = 10<br>PSP = 10<br>NC = 10 | prefrontal cortex<br>cerebellum | RIN, Age, PMI<br>None of the viable factors<br>showed significant<br>correlation to the gene<br>expression. | RefFinder | [6] |

Abbreviation：RG, reference gene; PMI, post-mortem interval; RIN, RNA integrity number; NC, non-demented control; AD, Alzheimer's disease; PD, Parkinson’s disease; ALS, Amyotrophic lateral sclerosis; HTD, Huntington’s disease; MS, Multiple sclerosis; SCZ, Schizophrenia; DLB, Dementia with Lewy bodies; MSA, Multiple System Atrophy; PSP, Progressive Supranuclear Palsy.

**References for Table S3**


1. Gutala RV, Reddy PH. The use of real-time PCR analysis in a gene expression study of Alzheimer's disease post-mortem brains. *J Neurosci Methods*. 2004;132(1):101-107. doi:10.1016/j.jneumeth.2003.09.005

2. Barrachina M, Castaño E, Ferrer I. TaqMan PCR assay in the control of RNA normalization in human post-mortem brain tissue. *Neurochem Int*. 2006;49(3):276-284. doi:10.1016/j.neuint.2006.01.018

3. Coulson DT, Brockbank S, Quinn JG, et al. Identification of valid reference genes for the normalization of RT qPCR gene expression data in human brain tissue. *BMC Mol Biol*. 2008;9:46. Published 2008 May 6. doi:10.1186/1471-2199-9-46

4. Leduc V, Legault V, Dea D, Poirier J. Normalization of gene expression using SYBR green qPCR: a case for paraoxonase 1 and 2 in Alzheimer's disease brains. *J Neurosci Methods*. 2011;200(1):14-19. doi:10.1016/j.jneumeth.2011.05.026

5. Durrenberger PF, Fernando FS, Magliozzi R, et al. Selection of novel reference genes for use in the human central nervous system: a BrainNet Europe Study. *Acta Neuropathol*. 2012;124(6):893-903. doi:10.1007/s00401-012-1027-z

6. Rydbirk R, Folke J, Winge K, Aznar S, Pakkenberg B, Brudek T. Assessment of brain reference genes for RT-qPCR studies in neurodegenerative diseases [published correction appears in Sci Rep. 2020 Jul 23;10(1):12559]. *Sci Rep*. 2016;6:37116. Published 2016 Nov 17. doi:10.1038/srep37116
